# Supplementary material for: The Ameliorative Effect of Dexamethasone on the Development of Autoimmune Lung Injury and Mediastinal Fat-Associated Lymphoid Clusters in an Autoimmune Disease Mouse Model
Source: Int J Mol Sci. 2022 Apr 18;23(8):4449. doi: 10.3390/ijms23084449 (PMC9027674; doi:10.3390/ijms23084449)
Supplement: Supplementary file 1 [file ijms-23-04449-s001.zip › ijms-1640196-supplementary.pdf]

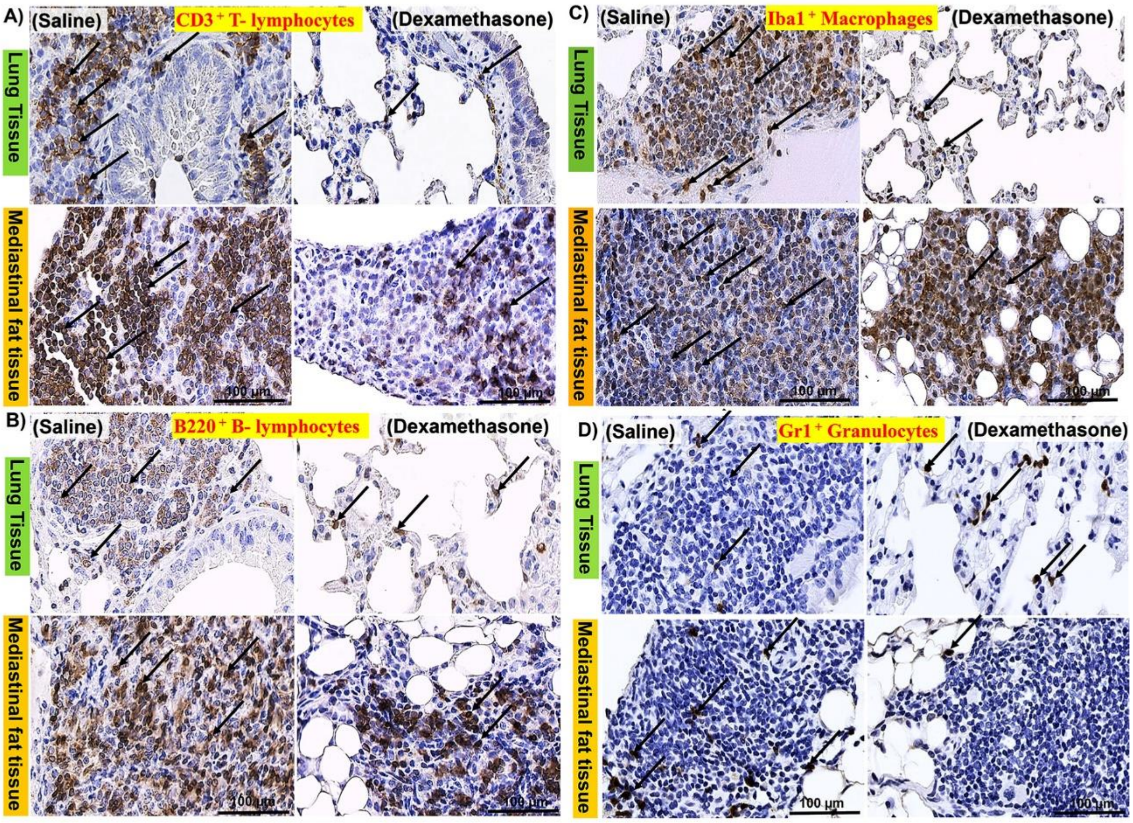

Figure S1. Representative photomicrograph of the immune cell populations in the lung, and mediastinal fat tissues of saline and dexamethasone groups in female Lpr mice. (A-D) Immunohistochemical-staining of the lung and mediastinal fat tissue of saline and dexamethasone groups for detection of $CD3^+$ T-lymphocytes (A), $B220^+$ B-lymphocytes (B), Iba-1^+ macrophages (C), and Gr-1^+ granulocytes (D). Notice immune-positive cells (arrows).

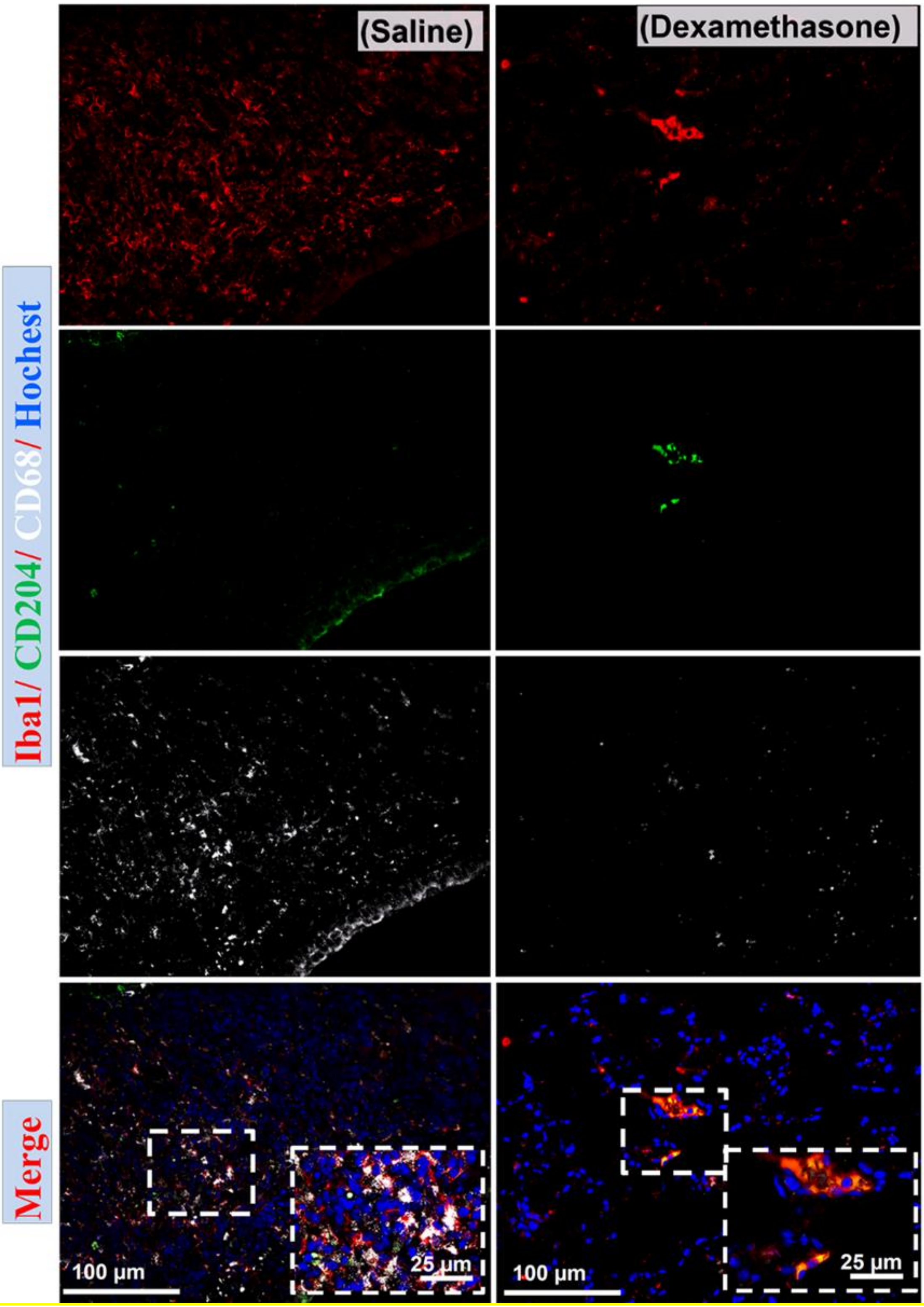

Figure S2. Representative photomicrograph of the macrophage's subsets, in the lung tissues of saline and dexamethasone groups in female Lpr mice. Triple immunofluorescent staining of Iba-1⁺ macrophages (red), CD86⁺ M1 (white) and CD204⁺ M2 (green) in the lung of saline and dexamethasone groups. Notice merged photograph with Hoechst nuclear staining (blue).

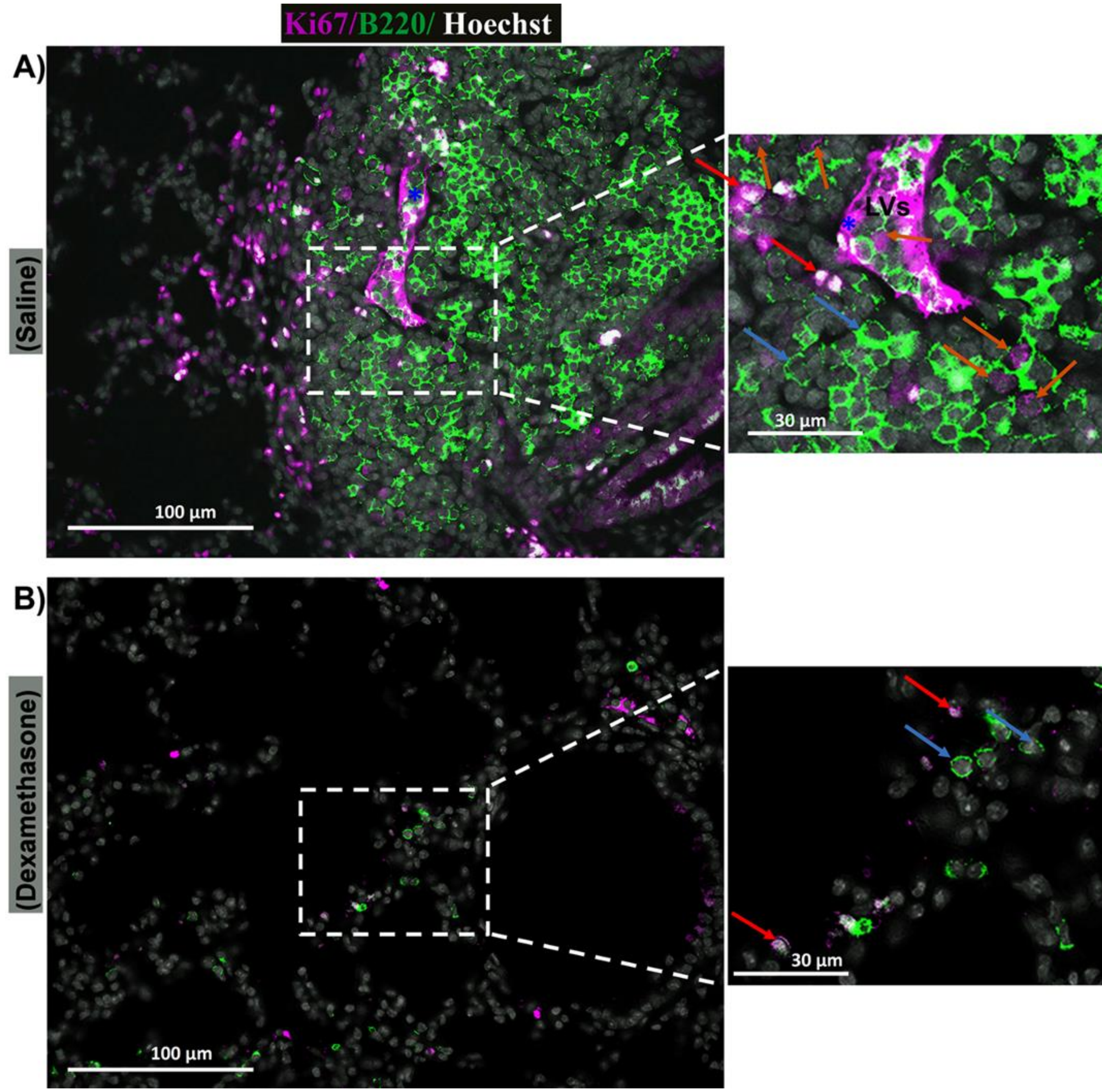

Figure S3. Representative photomicrograph of the co-staining for Ki67⁺ proliferating cell and the B220⁺ B-lymphocytes, in the lung tissues of saline and dexamethasone groups in female Lpr mice. Double immunofluorescent staining of Ki67⁺ proliferating cell (magenta), and B220⁺ B-lymphocytes (green) in the lung of saline and dexamethasone groups. Nuclear staining with Hoechst (white). The merged photographs revealed B220⁺ B-lymphocytes (blue arrows), Ki67⁺ proliferating cells (red arrows), Ki67⁺ B220⁺ proliferating B-lymphocytes (orange arrows). Notice lymphatic vessel (LVs) engorged with lymphocytes and their lining endothelial cells were Ki67⁺ (blue asterisk).

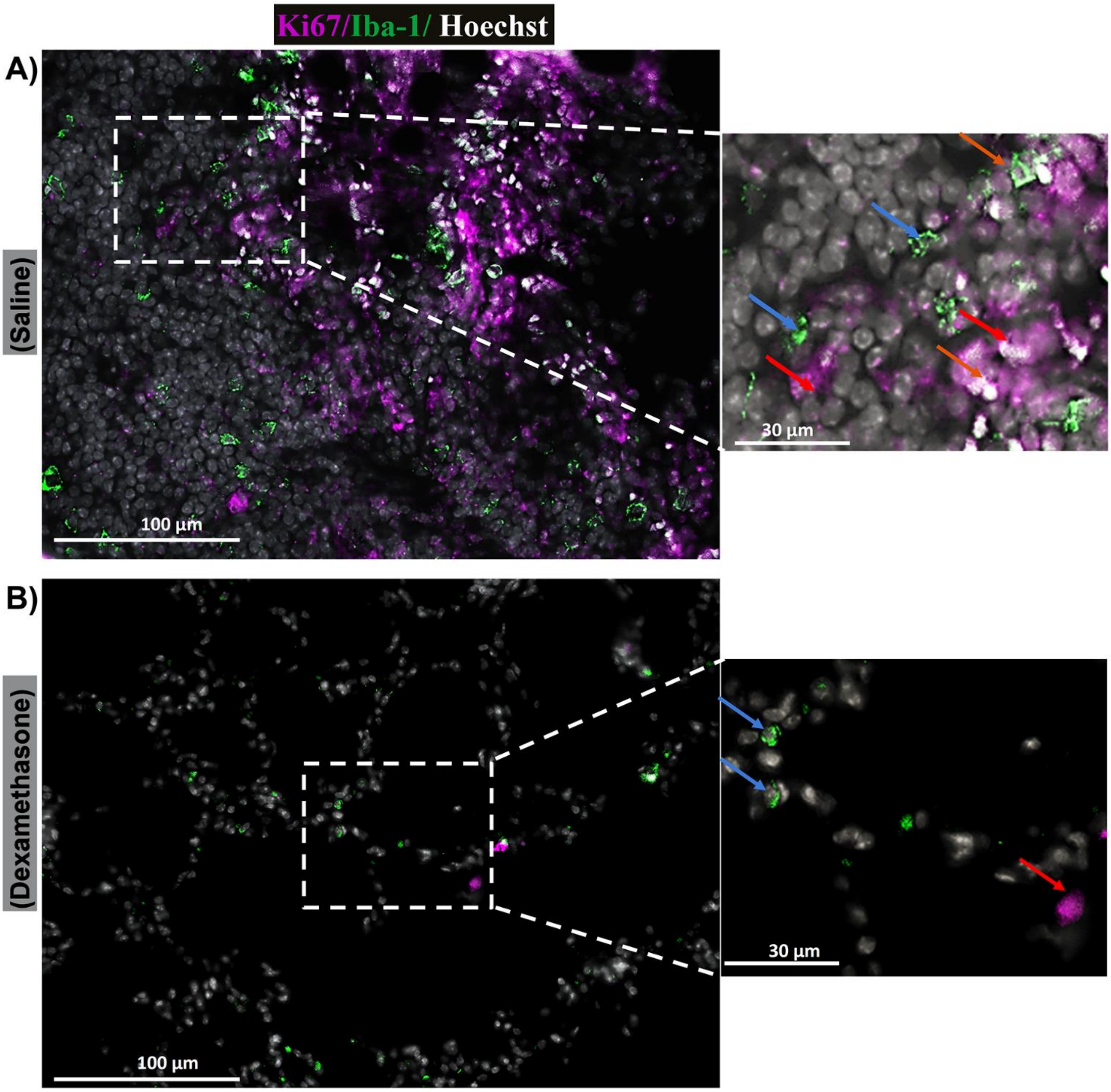

Figure S4. Representative photomicrograph of the co-staining for Ki67⁺ proliferating cell and the Iba-1⁺ macrophages, in the lung tissues of saline and dexamethasone groups in female Lpr mice. Double immunofluorescent staining of Ki67⁺ proliferating cell (magenta), and Iba-1⁺ macrophages (green) in the lung of saline and dexamethasone groups. Nuclear staining with Hoechst (white). The merged photographs revealed Iba-1⁺ macrophages (blue arrows), Ki67⁺ proliferating cells (red arrows), Ki67⁺ Iba-1⁺ proliferating macrophage (orange arrow).

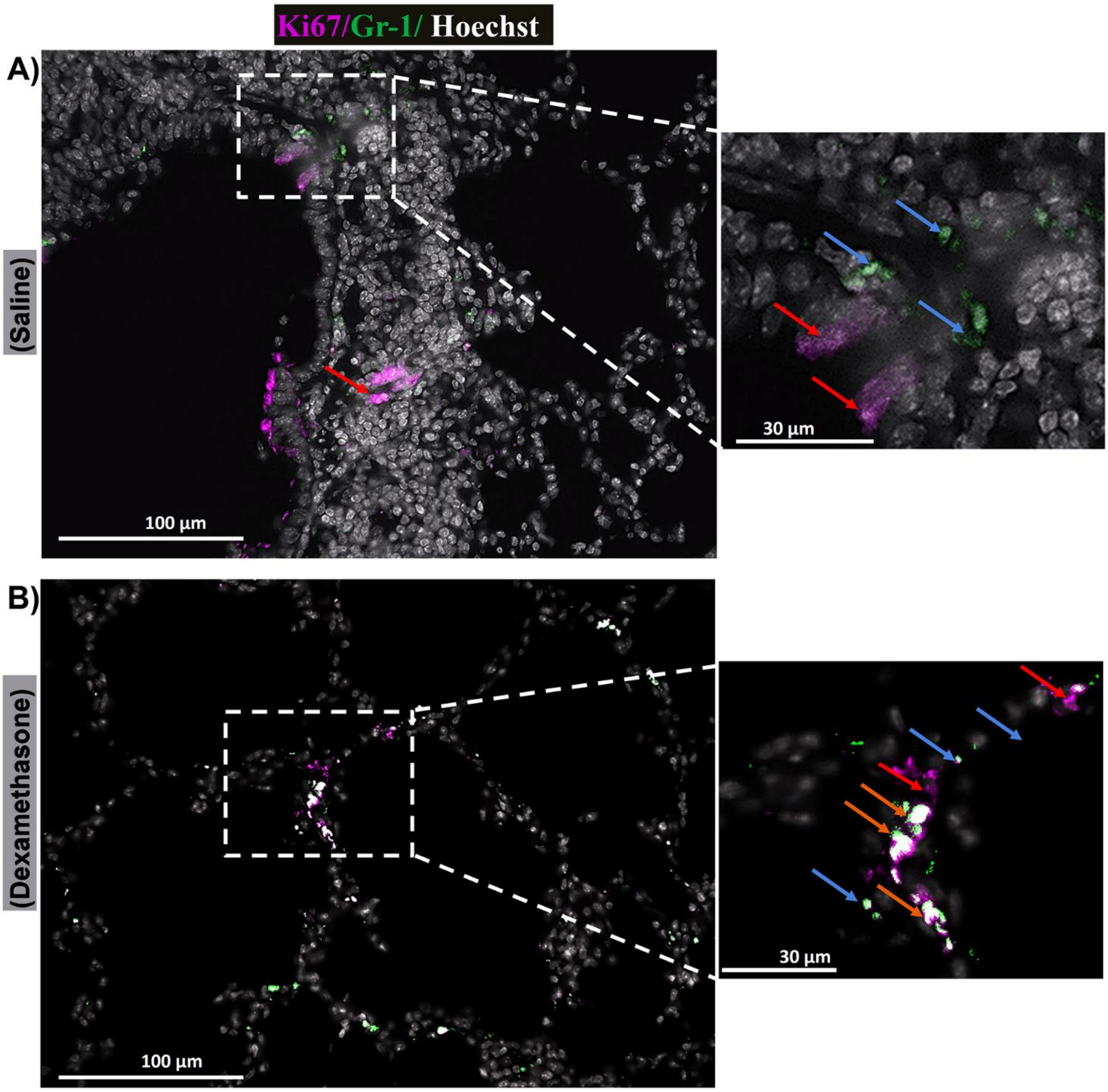

Figure S5. Representative photomicrograph of the co-staining for Ki67⁺ proliferating cell and the Gr-1⁺ macrophages, in the lung tissues of saline and dexamethasone groups in female Lpr mice. Double immunofluorescent staining of Ki67⁺ proliferating cell (magenta), and Gr-1⁺ granulocytes (green) in the lung of saline and dexamethasone groups. Nuclear staining with Hoechst (white). The merged photographs revealed Gr-1⁺ granulocytes (blue arrows), Ki67⁺ proliferating cells (red arrows), Ki67⁺ Gr-1⁺ proliferating granulocytes (orange arrows).

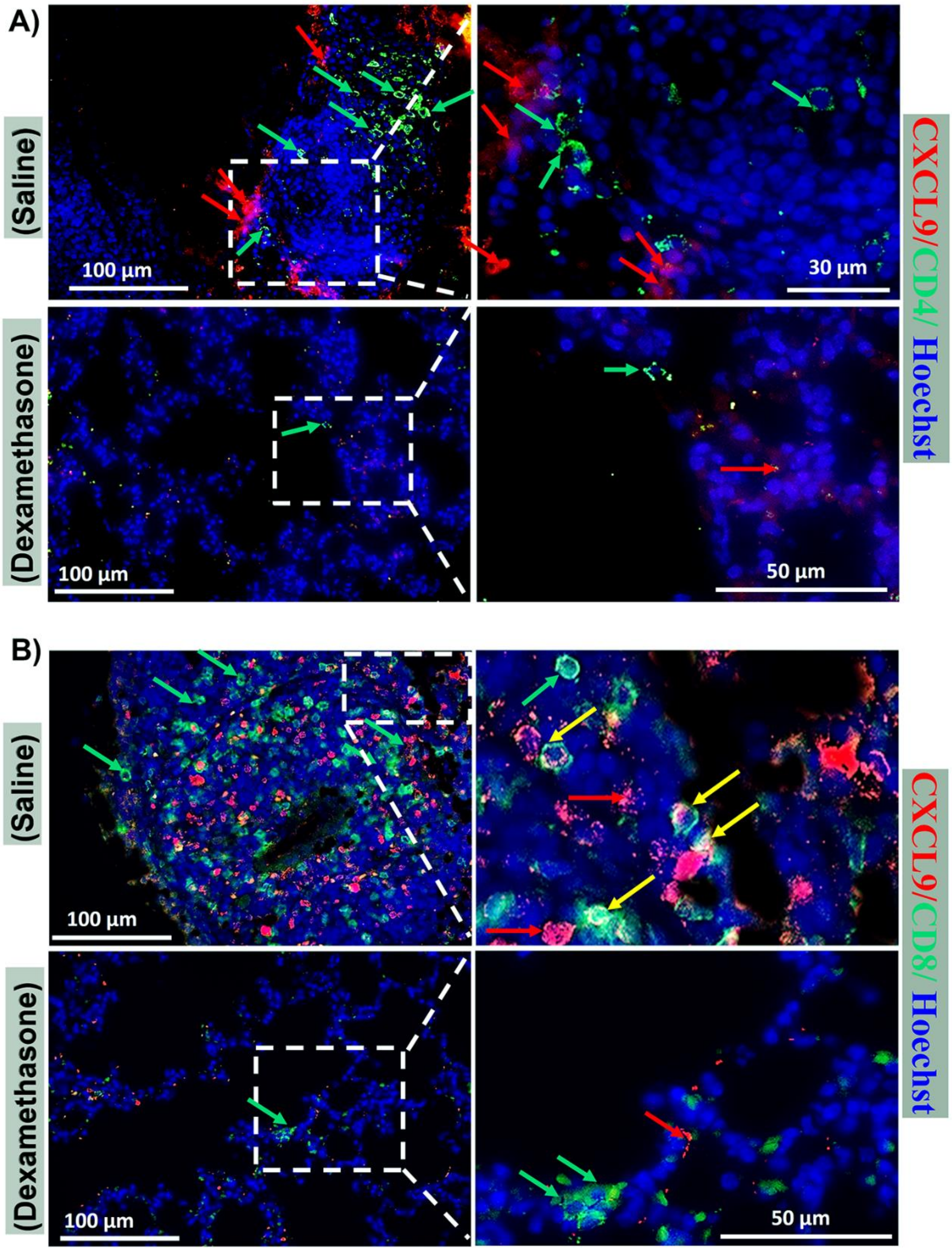

Figure S6. Analysis of the effect of dexamethasone on the expression of CXCL9 along with either CD4 or CD8 positive T-lymphocytes in the lung of female Lpr mice. (A-B) Representative merged images of double immunofluorescent staining for anti-CXCL9 (red) with either anti-CD4 "green" **(A)** or anti-CD8 "green" **(B)** antibodies, and Hoechst nuclear staining (blue). Notice positive reaction for CXCL9 (red arrows), CD4/ CD8 (green arrows), and CXCL9^{+} / CD8^{+} co-stained cells (yellow arrows).
